# Reduction of Oxygen Production by Algal Cells in the Presence of O-Chlorobenzylidene Malononitrile

**DOI:** 10.3390/bioengineering11060623

**Published:** 2024-06-18

**Authors:** Viorel Gheorghe, Catalina Gabriela Gheorghe, Daniela Roxana Popovici, Sonia Mihai, Raluca Elena Dragomir, Raluca Somoghi

**Affiliations:** Chemistry and Chemical Engineering Department, Petroleum—Gas University of Ploiesti, 39 Bvd. Bucuresti, 100520 Ploiesti, Romania

**Keywords:** CS gas, O-chlorobenzylidene malononitrile, ecotoxicity, chlorophyll, FTIR, SEM spectroscopy

## Abstract

Chemical compounds, such as the CS gas employed in military operations, have a number of characteristics that impact the ecosystem by upsetting its natural balance. In this work, the toxicity limit and microorganism’s reaction to the oxidative stress induced by O-chlorobenzylidenemalonitrile, a chemical found in CS gas, were assessed in relation to the green algae *Chlorella pyrenoidosa*. A number of parameters, including the cell growth curve, the percent inhibition in yield, the dry cell weight, the percentage viability and productivity of algal biomass flocculation activity, and the change in oxygen production, were analyzed in order to comprehend the toxicological mechanisms of O-chlorobenzylidenemalonitrile on algal culture. Using fluorescence and Fourier transform infrared spectroscopy (FTIR), the content of chlorophyll pigments was determined. The values obtained for pH during the adaptation period of the *C. pyrenoidosa* culture were between 6.0 and 6.8, O_2_ had values between 6.5 and 7.0 mg/L, and the conductivity was 165–210 µS/cm. For the 20 µg/mL O-chlorobenzylidenemalonitrile concentration, the cell viability percentage was over 97.4%, and for the 150 µg/mL O-chlorobenzylidenemalonitrile concentration was 74%. The EC_b50_ value for *C. pyrenoidosa* was determined from the slope of the calibration curve; it was estimated by extrapolation to the value of 298.24 µg/mL. With the help of this study, basic information on the toxicity of O-chlorobenzylidenemalonitrile to aquatic creatures will be available, which will serve as a foundation for evaluating the possible effects on aquatic ecosystems. The management of the decontamination of the impacted areas could take the results into consideration.

## 1. Introduction

One type of tear gas that is frequently used for riot control in military operations, law enforcement, and military personnel training exercises is CS gas [1,2,3,4]. In order to support investigations into claims of CWA use under the Chemical Weapons Convention (CWC), it is critical to determine whether an individual has been exposed to chemical warfare agents (CWAs) [5,6,7,8]. 

CS gas is the name of the chemical compound that contains the irritating substance O-Chloro benzylidene malononitrile. In our tests, we will call it CBM. O-chlorobenzylidene malononitrile is also known by other names, such as 2-chlorophenyl-methylene propanedinitrile, β, β-dicyano-o-chlorostyrene, and 2-chlorobenzal malonitrile. As with other xenobiotics, the long-term effects of chronically toxic chemicals of riot control agents disrupt the ecological balance of biocenoses. Due to the fact that this substance is used in crowd control operations, it is necessary to know the toxicological effects it generates on human health and the impact on the environment [9].

CBM is typically combined with a pyrotechnic substance and disseminated in grenades or canisters as fine particles that produce a distinctive smoke. These can be found in single containers, big bombs, or can be spread using a portable aerosolizer [10,11].

The European Parliament and Council of 23 October 2000 established the European Community directive 2000/60/EC, which established a framework for community action in the field of water policy and its later updates, 2006/11/EC and 2008/32/EC [12]. Every body of water needs to be safeguarded and maintained. Protecting ecosystem biodiversity is necessary to ensure the survival of all aquatic organism species, and as a result, quality with regard to ecotoxicological characteristics is also required [13].

Having these perspectives, a detailed knowledge of the mechanism of action of this substance is necessary in order to be able to develop enzymatic biodegradation methods using microorganisms that naturally live in aquatic environments and that do not require special procedures for their preservation/protection. CBM is a substance that could be easily removed from contaminated environments using certain microorganisms that are capable of enzymatically biodegrading this substance or its metabolic products. Despite continued recommendations against the use of CS gas over the past 20 years, it continues to be the most commonly used agent worldwide [14,15]. 

Chemically, 2-chlorobenzyl malononitrile (CSH2), 2-chlorobenzaldehyde (o-CB), 2-chlorohippuric acid, and thiocyanate are produced from CBM. Following inhalation exposure, CBM and its metabolites can be found in the blood [16]. Two of the metabolites of CS, 2-chlorobenzaldehyde and 2-chlorobenzyl malononitrile, were found in the blood of rodent and non-rodent animal species after they inhaled the chemical [17]. 

Following the research conducted by the National Institutes of Occupational Safety and Health (NIOSH) and Occupational Safety and Health Administration (OSHA), it was established that the safety limit for exposure to CBM at 0.4 mg/m^3^ and the value of immediate danger to life and health (IDLH) at 2 mg/m^3^. The lethal level of cyanide in the blood is 1 mg/L, and a concentration between 0.2 and 0.25 mg/L is considered toxic, meaning that it generates serious health conditions [18].

By adding particular microorganisms with a high tolerance to the hazardous toxin CBM to the aquatic flora, the toxicity of the material could be minimized, provided that exposure to it is limited [19].

During military operations that use large amounts of CBM, the handling of this substance also involves environmental pollution. In this study, we do not focus on the medical problems of people involved in conflicts. The purpose of our research is to find methods to guide the management of the pollutant in the case of exceeding certain concentration thresholds that could degrade the aquatic ecosystem. Through the research undertaken, results were obtained that can help the management of contaminated sites by supplementing the aquatic biofauna with microalgae or with microorganisms that are able to biodegrade toxic compounds [20].

This research is useful for establishing the ecotoxicity of special toxicants and assessing the ecotoxicological risk for contaminated sites, measures that will be able to be introduced in biomonitoring programs, such as those introduced through the Romanian Law no. 74/2019 regarding the management of potentially contaminated sites and contaminated sites which was published in the Romanian Official Gazette, Part I, no. 342 of 3 May 2019.

Through the performed tests, we aimed to study the toxicity of CBM, a substance that is used as a weapon to counter riots and in military operations. In earlier research, we assessed the capacity of specific microbes to break down CBM and determined the toxicity values of acute lethal concentrations (LC50) that can be utilized to ascertain the upper limit of acceptable concentrations that prevent the growth of typical fish fauna in contaminated waters. For an average concentration of 2.9 mg/L of CBM, the LC50 estimated at 24 h with a 95% confidence interval is 1.46, and for an average concentration of 1.2 mg/L of CBM, the LC50 estimated at 72 h with a 95% confidence range is 1,079 [21].

Microalgae are sensitive indicators of environmental changes, and due to the fact that they can survive and develop in freshwater and marine ecosystems, they can be used for environmental risk assessment because they have the ability to transform substances containing the elements nitrogen and phosphorus from contaminated waters into biomass and bioproducts [22,23]. The pH is an important parameter that influences the biodegradability of toxic substances by microorganisms. Toxic substances act as inhibitors of cellular enzymes or react with groups of proteins denaturing enzymes [24,25]. The transformations through which the microorganisms degrade the toxic substances in the final degradation products are aerobic or anaerobic decomposition (in the presence of oxygen or without oxygen) and anoxic decomposition (in the presence of nitrate ion) [26,27,28].

In our earlier investigations, we conducted toxicological analyses of the compound CBM on a variety of microorganisms, and we found that the EC_50_ for *Saccharomyces* sp. culture was around 0.25 mg/mL, while the EC_b50_ for *Chlorella* sp. culture was 0.44 mg/mL.EC_b50_ for *Lactobacillus* sp. culture was approximately 0.3 mg/L. Regarding the *Paramecium* sp. culture, the most susceptible to the effects of CBM was the culture with an EC_b50_ value of 50 μg /mL [19,29,30].

Enzymatic mechanisms occurring in the cells of microorganisms can lead to the elimination of toxic chemical substances from the petroleum industry [31,32,33,34], such as naphthenic acids and surfactants [32,35]. The toxic effects of certain chemicals generate enzymatic changes in algae cells, making it necessary to study certain enzymes influenced by chemical stress, including peroxidases (Px), superoxide dismutase (SOD), catalase (CAT), and glutathione reductase (GR) [36,37,38,39]. Considering this principle, in our previous tests, we studied the removal of metabolites after the hydrolysis of the substance CBM by using the algal suspension, and the results indicated that the suspension of *Chlorella* sp. consumed the entire amount of CBM from the samples [19,40,41]. 

## 2. The Toxicity Analysis

**Scheme 1:**
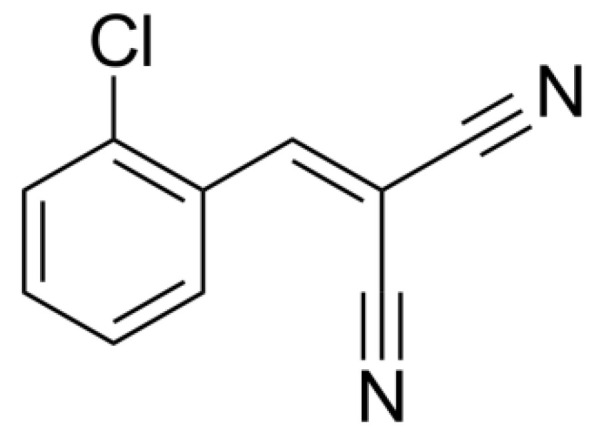
The chemical structure of o-chlorobenziliden malononitrile.

The molecular weight of O-chlorobenzyliden malononitrile (CBM) (Scheme 1) is 188.6 g/mol, and its water solubility is 2.0 × 10^−4^ M. Its chemical formula is C_10_H_5_ClN_2_. With the Cl atom in the ortho position and monosubstitution in the nucleus, CBM is an aromatic alkyl nitrile. It is an o-chlorostyrene derivative [25]. o-chlorobenzaldehide (C_7_H_5_ClO CAS 89-98-5, MW 140 g/mol), malononitrile (C_3_H_2_N_2_ CAS 109-77-3, MW 66 g/mol), and diethylamine (C_4_H_11_N, MW 73 g/mol, CAS Registry Number 109-89-7) were combined in an in-house procedure to create CBM. 

In a neutral aqueous environment, CBM is comparatively resistant to hydrolysis. For this reason, the amount of CBM in water has a different half-life depending on the pH of the environment. Thus, CBM is reduced by 50% in 14 min at pH 7.4 and 25 °C or within 0.17 min at pH 11.4 and 25 °C. In contrast, in an acidic pH environment (1–4), CBM becomes stable [11]. Certain investigations have determined that CBM hydrolysis happens in 635 min at 30 °C in aqueous conditions, but it speeds up in an alcoholic environment. For instance, in an alcoholic setting with 95% ethanol and 5% water, hydrolysis happens after 95 min at 30 °C and after 40 min at 40 °C. When the double ethylene link is broken by hydrolysis, malonic nitrile and 2-chlorobenzaldehyde are produced [25].

## 3. Materials and Methods

### 3.1. Experimental Design

A range of parameters were examined, including flocculation activity (FA), changes in oxygen production (DX), and examination of the chlorophyll pigment content, in order to comprehend the toxicological mechanisms of CBM on the culture.

The studies were carried out in two stages. The first stage was the adaptation of the algae culture to the working conditions in the laboratory and the promotion of cell growth up to an optimal concentration of cell development. The second stage of the tests consisted of a series of experiments to observe the evolution of the algal culture under chemical stress conditions by adding different concentrations of CBM to the reaction medium and comparing the resulting values with a control culture treated under the same conditions of work but without the toxic substance. 

In earlier experiments, we examined the proliferation of microorganisms in *C. pyrenoidosa* algae by measuring their rate of growth as a measure of their capacity to respond to hazardous stimuli and their rate of rise in cell concentration over time [25]. In continuation of these studies, we evaluated the chlorophyll content of *C. pyrenoidosa* developed in chemical stress generated by different concentrations of CBM, and we followed the absorption spectra and the evaluation of the chlorophyll fluorescence contained in *C. pyrenoidosa* developed in bioreactors.

After the hazardous substance treatment, Fourier transform infrared spectroscopy (FTIR) was used to analyze the content of chlorophyll pigments, fluorescence spectroscopy was used to analyze the surface structure of algal cells under chemical stress, and scanning electron microscopy (SEM) was used to analyze the surface structure of the algal cells.

### 3.2. Biological Medium and Algae Cells

Microalgae *C. pyrenoidosa* was cultivated in Erlenmeyer flasks containing specific algae growth medium (SAGM):250 cm^3^ of distilled water, MgSO_4_•7H_2_O (0.3 g), KNO_3_ (0.4 g), CaCl_2_ (0.4 g), NaH_2_PO_4_•2H_2_O (0.3 g), FeSO_4_•7H_2_O (0.02 g), NaNO_3_ (0.3 g), NH_4_Cl (0.2 g); pH was adjusted to 6.5. The algal concentration used for starting cellular growth was 5 g/L of dry cells dissolved in SAGM medium. *C. pyrenoidosa* microalgae was chosen for this study due to the fact that it is easy to cultivate, which makes it cost-efficient. It has a high growth speed and a high tolerance to chemical pollutants. The culture was obtained from the Culture Collection of Algae of Petroleum-Gas University of Ploiesti [16,25].

Erlenmeyer flasks (3 replications) were placed in a laboratory shaker (Orbital Multi-Shaker) at 100 rpm, with fluorescent light (range 60–120 µE∙m^−2^·s^−1^). The strains were maintained at 30 °C with a 12 h day/night photoperiod. Chemical reagents were weighed with an Ohaus model AX224M analytical balance. The laboratory experiments were conducted using a high-performance multi-parameter WTW Inolab Multi 9630 IDS, which has three galvanically isolated measuring channels for detecting conductivity, pH, and oxygen. Blind stoppers (gauze and cotton wool stoppers) were installed in the bioreactors, and they remained in place for the duration of the testing. The assessment of culture growth was conducted using the spectrophotometric method, which involved measuring the optical density (OD600) at 30-day intervals. Additionally, aliquot volumes were taken on a regular basis to measure the optical density and determine cell viability, dry cell weight (DCW), biomass productivity, and flocculation activity (FA) [22,27].

To determine these parameters, four replications performed simultaneously were analyzed, and the results obtained were processed as the arithmetic mean of the determinations. Growth was measured at 600 nm (optical density) and then converted into a unit of biomass (cells/mL) [28]. To analyze the purity of the algal culture and the appearance of the cells, a Celestron Microscope, model 4434, equipped with a Thoma Marienfeld, was used. 

Following the measurement of cell viability, Equation (1) was used to compute the average specific growth rate (µ), which is an expression referring to the logarithmic increase in biomass throughout the exposure period.

µ = (lnN_n_ − lnN_0_)/(t_1_ − t_n_), day^−1^ (1)

t_1_ = is the time at the start of the test where the values are represented by measurements performed at 30 days (t_n_); N_0_ represents the number of cells/mL measured at the beginning of the test; N_n_ represents the number of cells/mL measured at t_n_.

Biomass productivity was calculated from Equation (2) considering the dry cell weight.

BP = (DCW_2_ − DCW_1_)/(t_2_ − t_1_), mg/L (2)

where DCW is the biomass weight at the beginning of the test t_1_ and, respectively, at the time of the test t_2_.

The flocculation activity (FA) was calculated from Equation (3).

FA = (A − B) A × 100, %(3)

where A = Absorbance of the algal culture at 600 nm at the start in the control and B = Absorbance at 600 nm of the algal culture after 30 days of cellular growth [28].

### 3.3. Preparation of CBM Concentrations

During the second testing phase, solutions containing varying concentrations of CBM were prepared by dissolving them in water. These concentrations were C1 (20 µg/mL), C2 (40 µg/mL), C3 (60 µg/mL), C4 (80 µg/mL), C5 (100 µg/mL), C6 (120 µg/mL), and C7 (150 µg/mL). The goal was to assess the toxicity of CBM on the culture of Chlorella pyrenoidosa. Using a Soltec-type Ultrasonic SONICA S3 ultrasonic dispersion apparatus, the CBM concentrations were created.

### 3.4. Preparation of the Bioreactors with Biological Samples Contaminated with CBM for Testing the Dissolved Oxygen

Two series of test tubes labeled ABCBM series and ACBM series were used for testing. In the first series marked ABCBM, 2 mL (10^4^ cells/mL) of *C. pyrenoidosa* algae suspension in the exponential growth phase, labeled ABCBM01- ABCBM07 containing different concentrations of CBM (20 µg/mL), (40 µg /mL), (60 µg/mL), (80 µg/mL), (100 µg/mL), (120 µg/mL), (150 µg /mL). In the second series labeled ACBM, no algal suspension was added, containing different concentrations of CBM; the tubes were labeled as follows: ACBM01 (20 µg/mL), ACBM02 (40 µg/mL), ACBM03 (60 µg/mL), ACBM04 (80 µg/mL ml), ACBM05 (100 µg/mL), ACBM06 (120 µg/mL), and ACBM07 (150 µg/mL).

For the determination of dissolved oxygen, two containers were additionally prepared, containing the control with algal suspension 2 mL (10^4^ cells /mL), marked MCHL, and the control without algal suspension, marked M. The control contained distilled water. All the containers were incubated for 24 h, mechanically stirred in an orbital shaker (100 rpm), and were kept at a temperature of 30 °C under white light (intensity was in the range 60–120 µE∙m^−2^·s^−1^).

### 3.5. Determination of Oxygen Production of Algal Culture in Chemical Stress Generated by CBM

A high-performance multi-parameter WTW Inolab MULTI 9630 IDS with three galvanically isolated measuring channels for conductivity, pH, and oxygen measurement was used to determine the concentration of oxygen [29]. The results obtained regarding oxygen production (Dx) were processed and compared according to the mathematical relationship in Equation (4). The difference between the environments that contained *C. pyrenoidosa* and toxic algae was marked (*OX*) (ABCBM series) and compared with the corresponding sample with toxic but without algal suspension (ACBM series), which was marked (*Ox*). The obtained value was decreased from the difference in the oxygen concentration obtained from the value of the control with algal suspension MCHL, denoted (*OB*), and the control without algal suspension, *M*, marked (*Ob*).

(4) $$Dx=\left[\frac{\left(OX-Ox\right)-\left(OB-Ob\right)}{\left(OB-Ob\right)}\right]\times 100,\;\%$$

#### 3.5.1. The Percentage of Cell Growth Inhibition

According to the OECD Guidelines for the Testing of Chemicals of the Organisation for Economic Cooperation and Development (OECD), the inhibition ratio (IR) was analyzed by enumeration of cell numbers every 24 h under an optical microscope (×400). Equation (5) was utilized to determine the percentage of cell growth inhibition (% IR) at every concentration of the drug under examination.

IR = Cc − Ct/Ct × 100, %(5)

Cc, cell number density of control culture, cells/mL, Ct, cell number density of samples with specific concentrations, (20–150 mg/mL CBM), cells/mL. 

#### 3.5.2. The Percent Inhibition in Yield (%I) Calculated with Equation (6)

I = (YC − YR)/YC × 100, %(6)

where YC = value for yield in the control group, YR = value for yield for the treatment replicate. For each concentration of CBM tested, the yield was calculated as the difference between the biomass at the end of the test and the initial biomass for each series analyzed relative to the initial biomass.

### 3.6. Contents of Photosynthetic Pigments

#### 3.6.1. Preparation of Bioreactors for Chlorophyll “a” and Chlorophyll “b” Analysis

In order to analyze chlorophyll, test tubes with caps were prepared in 4 series of replicates. Each test tube contained 10 mL of liquid and was inoculated with 2 mL 10^4^ cells/mL of C. pyrenoidosa algae suspension in the exponential growth phase. Four test tubes were used as a control and were diluted with distilled water; four series that each contained seven test tubes in which a quantity of CBM was placed so that each container contained a specific concentration (20 µg/mL, 40 µg/mL, 60 µg/mL, 80 µg/mL, 100 µg/mL, 120 µg/mL, 150 µg/mL). 

In two series of replicates, the chlorophyll content was analyzed without being incubated, and the other two series of replicates were incubated for 24 h by mechanical shaking in an orbital shaker at a temperature of 30 °C with a photoperiod of 12 h day/night.

#### 3.6.2. Chlorophyll Quantification

The chlorophyll extract was obtained by centrifugation of the test tubes using a centrifuge Universal 320R Tip 1406-01 at 5000 rpm, 10 min. The supernatant was removed. To extract chlorophyll pigments, the cell pellets were re-suspended in 2 mL methyl alcohol 90%, followed by heating on the electric stove at 60 °C for 30 min. After this period, the suspension was centrifuged at 10,000 rpm for 10 min, and the supernatant was collected and measured at 652 nm and 665 nm using UV-VIS. The concentrations of chlorophyll “a” and “b” (Chl-a and Chl-b) were calculated and reported in µg/mL. 

For the accuracy of the results, the average values of the results obtained for each concentration were determined. The concentration of chlorophyll “a” and chlorophyll “b” were calculated using the following Equations no. (7) and (8) [31,32,36,37].

Chl-a = 16.29A_665_ − 8.54A_652_, (µg/mL) (7)

Chl-b = 30.66A_652_ − 13.58A_665_, (µg/mL) (8)

#### 3.6.3. FTIR and Fluorescence Analysis of the Chlorophyll Extract

After UV-VIS photometric measurements of the chlorophyll pigment, the same samples were scanned by FTIR using the TRACER IR spectrophotometer, Fourier transform infrared spectrophometer, and examined with the fluorescence spectrophotometer RF 6000 Spectro fluorophotometer Shimadzu.

## 4. Results and Discussion

**Figure 1:**
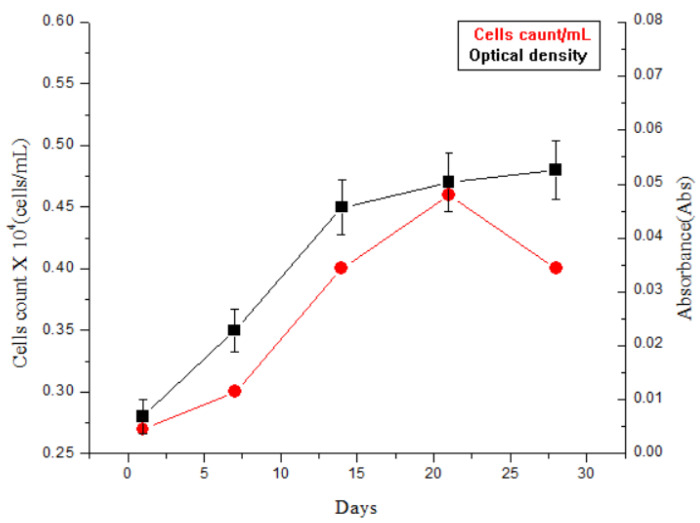
Evolution of cell growth (optical density) and viability (cells count/mL) of algal cells during the testing period. Error bars represent standard deviation (n = 4).

**Figure 2:**
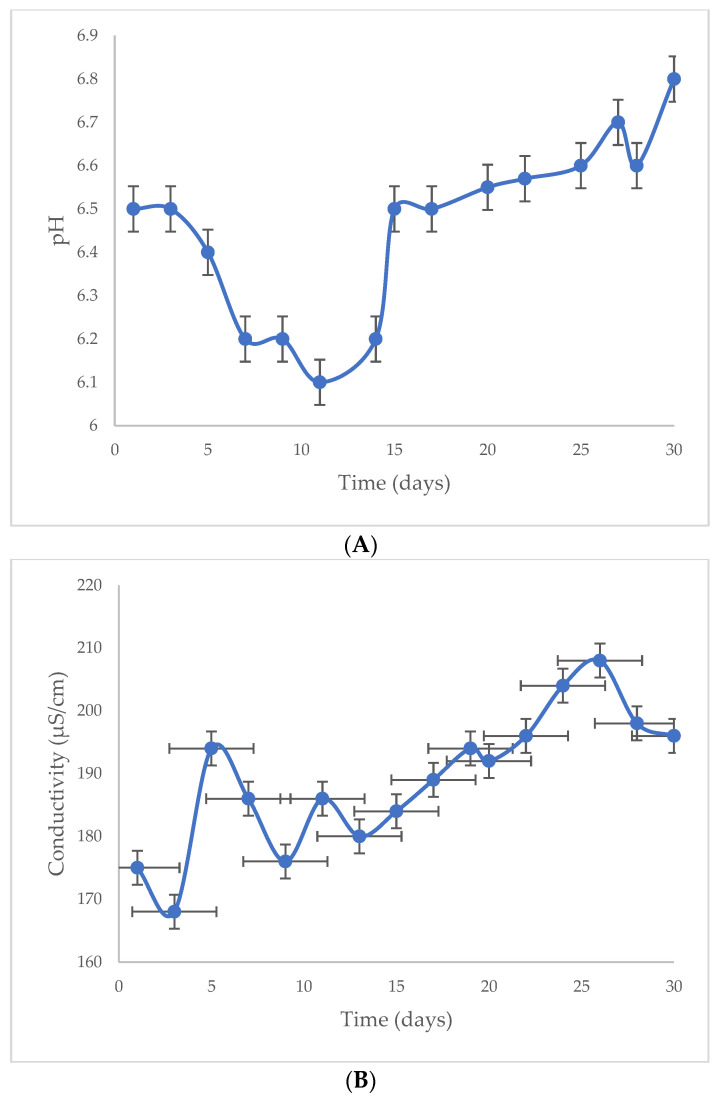
Evolution of pH (**A**) and conductivity (**B**) during the cell development period. Error bars represent standard error (**A**) and standard deviation (n = 4) (**B**).

To enhance gas exchange and lessen pH variation in test solutions, mechanical stirring was used to maintain the microorganisms in suspension during the testing process. Until day 9, cell growth was ascending, relatively small, after which a lag period followed until 13 days from the beginning of the experiment. The exponential phase began from day 12 to day 23. On day 27, the cellular growth was maximum; after this period, it entered a period of decline. (Figure 1). The pH had a decreasing evolution, reaching around the value of 6 approximately in the middle of the test period, after which it oscillated around the value of 6.8–6.5, and as for conductivity, it had values that oscillated between 165 and 210 µS /cm (Figure 2)

**Figure 3:**
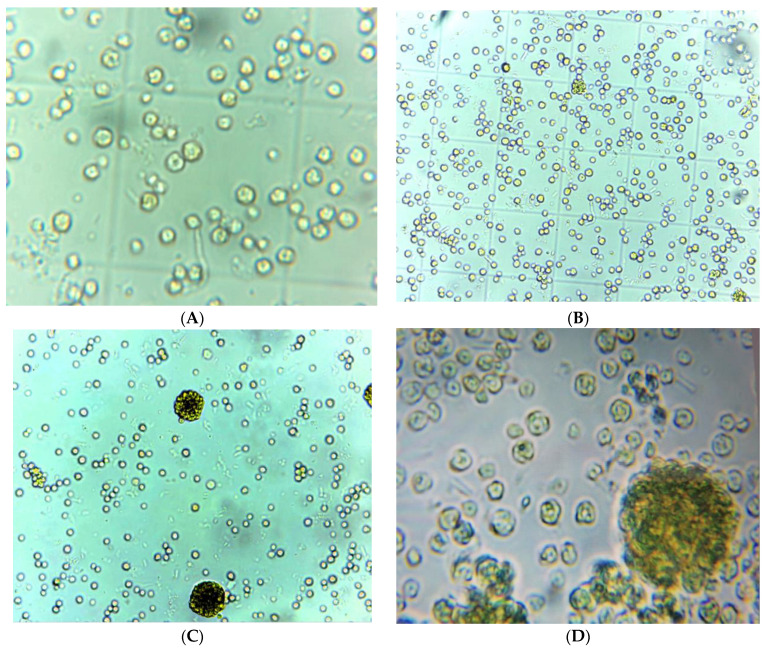
Microscopic image × 40 of *C. pyrenoidosa* strain in the period of cellular growth, visual results of biocoagulation in lag phase (**A**,**B**) cells of the algae *Chlorella pyrenoidosa*, in stationary phase (**C**,**D**).

The microscopic examination during the tests indicated good cellular development with the appearance of solitary cells in the first stage, and then cell agglomerations appeared due to the flocculation of the cells, this being favorable to the protection of the cellular membrane. (Figure 3). This would be useful for detoxification mechanisms in case of chemical stress through the synergistic enzymatic action of enzymes involved in cell synthesis [33,34,35,39,42,43].

During the tests, BP had a value of 11.31% after 15 days from the beginning of the tests and a value of 67% at the end of the test, after 30 days of cell growth, while the flocculation activity was approximately 80% [44,45].

**Figure 4:**
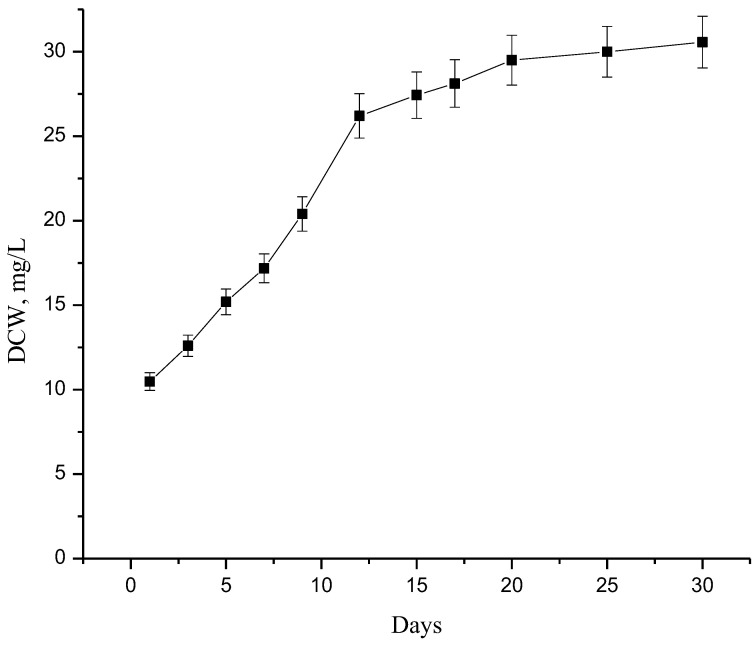
Time variation of dry cell weight (DCW). Error bars represent standard deviation (n = 4).

Algal draw weight (Figure 4) was measured using Macherey–Nagel filter no. MN 640 m dried at 95 °C for 24 h. The values obtained were from 10 mg/L after the first day of incubation, then increased to 25 mg/L after 15 days of incubation, and reached 30 mg/L at the end of the tests.

**Figure 5:**
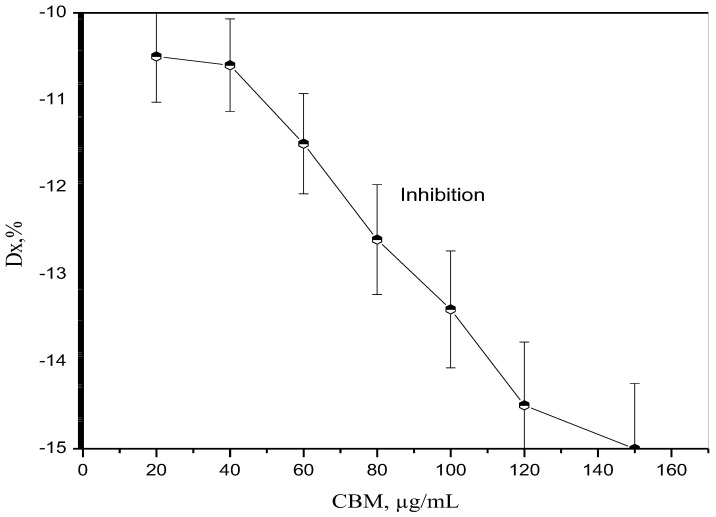
Oxygen production (Dx) of the algal culture *Chlorella pyrenoidosa.* Error bars represent standard deviation (n = 4).

The experimental results concerning the change in oxygen production regarding the concentration of algal suspension in different concentrations of CBM are presented in Figure 5. The aspect of the experimental curve obtained from the studies indicates that the substance CBM has a toxic action on *C. pyrenoidosa* algae; the descending curve of the representation Dx % underlines the fact that the percentage production of oxygen presents negative values and draws attention to the fact that the toxicity in the algal cell increases with increasing the concentration of CBM. Analyzing the obtained results, it is found that all the concentrations of CBM analyzed have an inhibitory action on the production of oxygen through the photosynthesis of the algae *Chlorella pyrenoidosa.*

**Figure 6:**
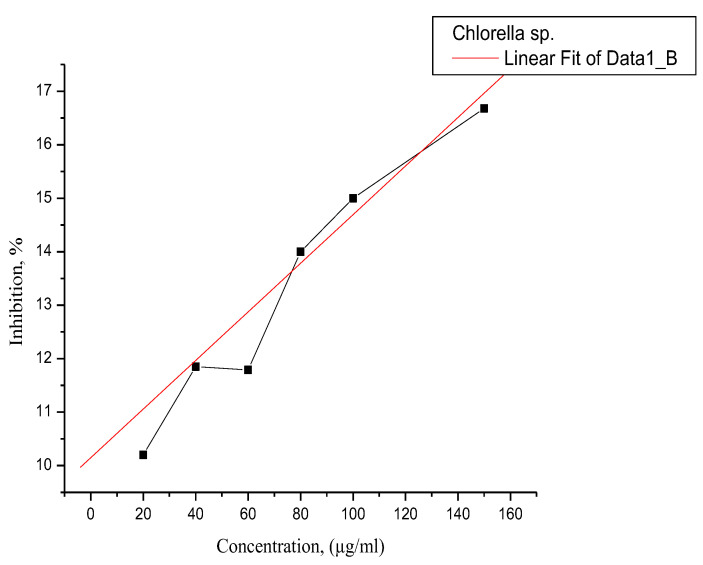
Cell inhibition on the algal suspension Chlorella pyrenoidosa. Error bars represent standard deviation (n = 4).

The percentage of inhibition having a negative value, the shape of the obtained curve indicates that the toxin influences the development of the algal suspension, implicitly photosynthesis. In the first phase, the curve is positioned vertically until the consumption of oxygen in the sample, the toxin having an algistatic action, after which, starting from the concentration of 40 µg/mL, the appearance of the curve is downward, the CBM’s action on the algal culture being algicidal. *C. pyrenoidosa* has a high tolerance to the pollutant, the inhibition measured by oxygen production is low, the obtained curve indicates a moderate toxic action, a concentration of 20 µg/mL induces a 10% cellular inhibition, while a concentration of 100 µg/mL induces an inhibition of cell growth of approximately 14%. With a 16% inhibition at 150 µg/mL, higher dosages do not significantly stop the growth of algal cells. (Figure 6). The percent inhibition in yield (%I) was calculated using Equation (6). Plotting the concentration that corresponds to 50% inhibition allowed for the estimation of the ECb50 value from the regression line. Based on the calibration curve’s slope, the EC_b50_ value for C. pyrenoidosa was calculated and extrapolated to a value of 298.24 µg/mL.

Analyzing the obtained results, we notice that the higher the concentration of the toxic substance, the lower the amount of dissolved oxygen, and the inhibition of cell growth increases with CBM concentration. Chlorella pyrenoidosa possesses chloroplasts of different shapes, with a granular appearance, green, ovoid shape, and slightly flattened, and they have the role of allowing the passage of light to the chlorophyll molecule, triggering the photosynthesis process. Chloroplasts contain chlorophyll pigments that represent pigments for light harvesting that participate in photosynthesis and that contain chlorophyll “a” and ‘b’.

In the presence of chlorophyll “a” and under light conditions, chloroplasts absorb light energy and transform it through catalytic systems into chemical energy. A role in photosynthesis is also played by carotenoids that transmit part of the light energy that they absorb to chlorophyll “a” and protect chlorophyll from photooxidation.

Chlorophyll “a” has the ability to receive light and initiate a chain of chemical reactions. During photosynthesis, chlorophyll pigments are not consumed but intervene only through a catalytic action. The absorption spectrum of pigment molecules shows that they absorb red light at 660 nm. Chlorophyll “a” is the biochemical parameter that is an indicator of the biomass that gives information about the content of nutritional elements, and the concentration of chlorophyll gives information about the stock of nutrients [41,43,46,47,48,49].

A variety of metabolic pathways can be used to break down chemicals, including the oxidation of carbon and hydrogen from organic materials, the oxidation of nitrogen from nitrites or from chemicals that contain nitrogen in their molecules, hydrolysis—the process of removing water at the carbon atom by adding it to a double bond—splitting and forming C-C bonds by decarboxylating or carboxylating ketones, and adding or removing the nitrogen atom in the form of NH3. Apart from the cellular metabolic processes, there are other reactions that lead to the inactivation and removal of hazardous organic compounds from the reaction medium, such as acetylation and methylation [44].

One way to assess possible photosystem damage is to look at a living system’s fluorescence. Chlorophyll (CHL) is used in these techniques as an internal probe of an organism’s ability to photosynthesize. 

Following the tests performed, the values obtained regarding the determination of the chlorophyll “b” content were between 0.27 µg/mL in the unincubated control sample and 1.6 µg/mL in the control sample incubated for 24 h, respectively, had a chlorophyll “a” content between 3.5 µg /mL and 8.5 µg/mL in the incubated control sample. The unincubated series had a content of chlorophyll “b” of 0.19 µg/mL and chlorophyll “a” 6.9 µg/mL for a concentration of 20 µg/mL CBM, chlorophyll “b” content of 0.012 µg/mL, respectively, chlorophyll “a” content 1.96 µg/mL for a concentration 150 µg/mL CBM. The series of incubated samples had a chlorophyll “b” content of 1.2 µg/mL, respectively, a chlorophyll “a” content of 8.5 µg/mL for a concentration of 20 µg/mL CBM and a chlorophyll “b” content of 0.05 µg/mL, respectively, chlorophyll “a” 2.09 µg/mL for a concentration of 150 µg/mL CBM. 

**Figure 7:**
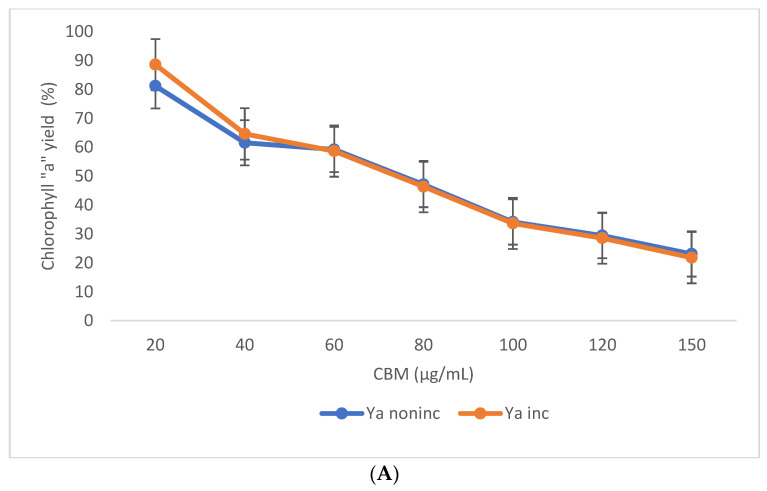

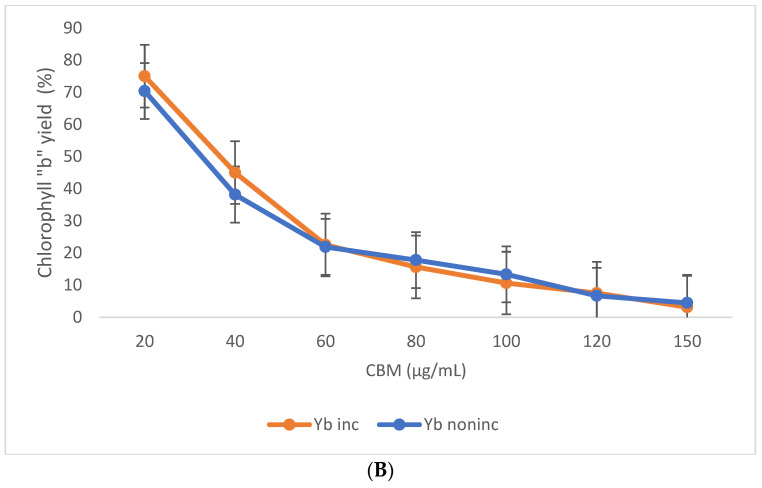
Chlorophyll yield (**A**) chlorophyll “a” Ya noninc/inc (non-incubated/incubated), (**B**) chlorophyll “b” Yb noninc/inc (non-incubated/incubated). Error bars represent standard deviation (n = 4).

The obtained results indicate that *C. pyrenoidosa* algae was inhibited by the presence of the toxic CBM. Therefore, considering the chlorophyll content as 100% in the control samples, we conclude that yield of *C. pyrenoidosa* in the bioreactor that had the C1 (20 µg/mL) CBM concentration, the chlorophyll “b” extraction yield was 70.3% for the non-incubated series and 75% for the 24 h incubated series, respectively, the chlorophyll “a” extraction yield was 81.1% for the non-incubated series and 88.5% for the 24 h incubated series. (Figure 7). In the bioreactor that had the C3 (60 µg/mL) CBM concentration, the chlorophyll “b” extraction yield was 21.8% for the non-incubated series and 22.5% for the 24 h incubated series, respectively, the chlorophyll “a” extraction yield was 59.1% for the non-incubated series and 58.65% for the 24 h incubated series.

In the bioreactor that had the (150 µg/mL) CBM concentration, the chlorophyll “b” extraction yield was 4.4% for the non-incubated series and 2.1% for the 24 h incubated series, respectively, the chlorophyll “a” extraction yield was 23.1% for the non-incubated series and 21.7% for the 24 h incubated series. In the non-incubated series, the presence of the toxicant inhibited the development of *C. pyrenoidosa* with a higher yield than the incubated series. We assume that incubation under conditions conducive to cell development favors cell regeneration, which is why the yield of cell inhibition in the presence of the toxicant is lower in the incubated series compared to the non-incubated series [11].

Diminished Overall Reflectance: The samples were characterized using Fourier transform infrared spectroscopy (ATR-FTIR), which has the potential to be used for determining the composition and biomass of cells from bioreactors (Figure S1) (provided in the additional material). A functional group was allocated to each peak [45,46,47,48]. Using an FTIR spectrophotometer (Shimadzu IR TRACER-100, Kyoto, Japan) in the 4000–400 cm^−1^ range, the surface chemistry of the materials was examined. 

In the cell walls, glycogen stores energy, and macromolecular makeup includes protein amide I band primarily (C=O) stretching 1583–1700 cm^−1^, protein as (−CH_2_) and as (−CH_3_) bending of methyl, 1425–1477 cm^−1^, nucleic acid (other phosphate-containing compounds) (>P=O) stretching of phosphodiesters 1191–1356 cm^−1^, carbohydrate (C-O-C) of polysaccharides 1134–1174 cm^−1^, carbonic acid (C-O-C) of polysaccharides stretching of phosphodiesters 1072–1099 cm^−1^ (>P=O) by nucleic acid and other phosphate-containing substances [49,50,51]. These include proteins (amide I and II), lipids (methyl and methylene groups, esters), carbohydrates (cellulose from 1100 cm^−1^ to 900 cm^−1^), pectin (bands at 1610 cm^−1^, 1424 cm^−1^, 960 cm^−1^), and cellulose (bands at 1162 cm^−1^, 1060 cm^−1^, 1030 cm^−1^), and esters in the carboxylic group (bands 1720–1700 cm^−1^); locations of protein amides I and II are visible at 1050 and 1012 cm^−1^ [25,45].

Figure S2 (presented in additional material) presents the graph obtained after the IR scan of CBM, 100 µg/mL aqueous solution. In the alcoholic environment, the hydrolysis reaction accelerates. Hydrolysis breaks the double ethylene bond with the formation of 2-chlorobenzaldehyde and malonic nitrile [25,43,44].

The carbon atoms of CBM are lost as malononitrile, which is partially metabolized to cyanide and thiocyanate, hydrolyzes to the carboxylic acid, and then undergoes an initial hydrolysis to 2-chlorobenzaldehyde and a reduction to 2-chlorobenzylmalononitrile (dihydro-CS) [47].

Following the metabolic reactions in the cell of *C. pyrenoidosa* and following the chemical reactions in the reaction medium, functional groups can result, which contain compounds C=NH−, −CH, −NH_2_, methyl and methylene groups, esters, from 1100 to 900 cm^−1^, carboxylic group of esters (bands 1720–1700 cm^−1^), (C=O) stretching 1583–1700 cm^−1^, (−CH_2_), and as (−CH_3_) bending of methyl 1425–1477 cm^−1^ [48,49,50,51,52,53].

In Figure S3 (presented in additional material), the FTIR graph is presented, in which section (A) shows a suspension of C. pyrenoidosa before extracting the chlorophyll pigment, in section (B), *C. pyrenoidosa* suspension in the control after the chlorophyll was extracted, presented a transmittance of 58%. In section (C), *C. pyrenoidosa* suspension in the presence of the toxic CBM (20 µg/mL), a transmittance of 59% was recorded, and in section (D), the analyzed sample was *C. pyrenoidosa* suspension in the presence of the toxic CBM (60 µg/mL) and indicated a transmittance of 64.%. In section (E), *C. pyrenoidosa* suspension in the presence of the toxic CBM (100 µg/mL) indicated a transmittance of 68.%, and in section (F), *C. pyrenoidosa* suspension in the presence of the toxic CBM (150 µg/mL) indicated a transmittance of 72%.

**Figure 8:**
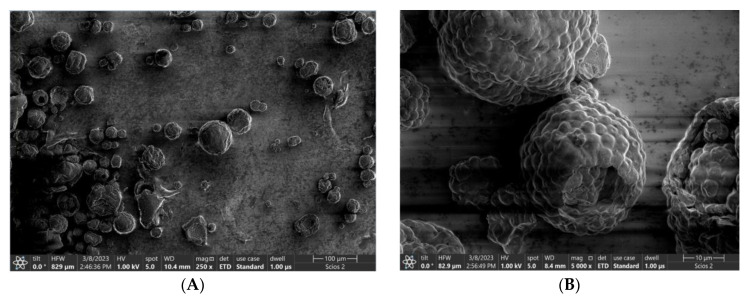

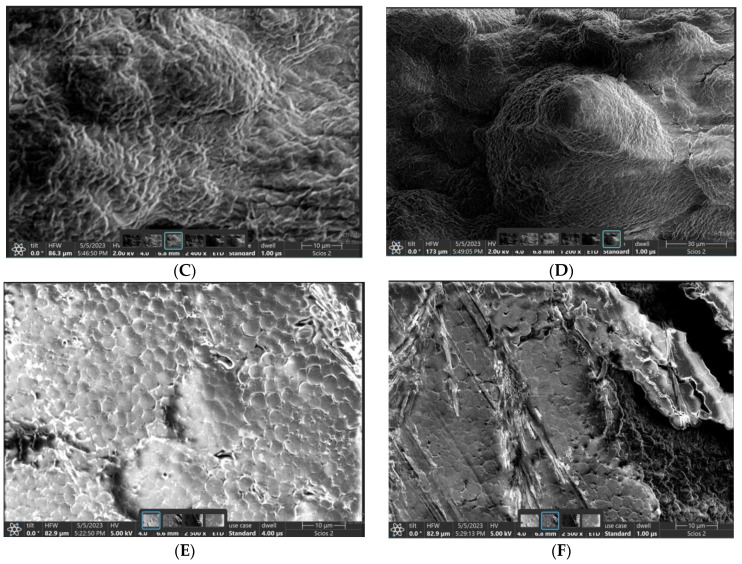
Record of a dry culture of the algae *C. pyrenoidosa* from a scanning electron microscope—Surface structure in control (algal grow (**A**) mag 250×, (**B**) mag 5000×), Strain culture with CBM) (150 µg/mL) after 24 h contact [(**C**) 2400× (60 µg/mL), (**D**) 1200 × and surface detail (**E**) and (**F**)].

Following the determination of chlorophyll content through the FTIR scanning of the samples, we noticed that as the concentration of chlorophyll in the sample decreases due to the cellular inhibition generated by the presence of the toxic CBM, the band at 1045 cm^−1^ increases, indicating an increasingly high transmittance. The interpretation we give is associated with the fact that the presence of the toxicant in the samples creates chemical stress and the photosynthesizing apparatus of *Chlorella pyrenoidosa* is destroyed, the chlorophyll pigment is found in a gradually lower concentration, and the fact that the peak increases around the corresponding value of carbohydrates (starch, cellulose from 1100 to 900 cm^−1^), we attribute this to the fact that the cell wall of the alga *C. pyrenoidosa* is destroyed due to the concentration gradient by the gradual addition of CBM. Thus, by centrifuging the sample in order to extract chlorophyll, components of the cell wall are also found in the analyzed suspension. The *C. pyrenoidosa* cell in the reaction medium metabolizes part of the toxic CBM, and following this process, in the sample could be found metabolic products that come from the conversion of CBM into 2-chlorobenzyl malonononitrile and 2-chlorobenzaldehyde. As the concentration of chlorophyll decreases due to the presence of the toxic CBM, the transmittance (T%) increases. The interpretation of this modification could be associated with the fact that it accumulates toxic agents in the analyzed sample. *C. pyrenoidosa* destroys toxic CBM and it accumulates metabolic products resulting from the metabolism of CBM [54,55,56,57,58,59]. Images regarding the appearance of the surface structure by SEM spectroscopy of *C. pyrenoidosa* in the presence of different concentrations of CBM are shown in Figure 8. The membrane remains intact, which means that enzyme activity in the reaction center is possible.

The addition of CBM in the reaction medium generates chemical stress in the bioreactors and affects the permeability of the cell by the fact that photosynthetic electron transport is blocked [56]. The chemical stress generated by CBM prevents the cellular development of the algae and destroys its enzymatic system, which generates cell death. *C. pyrenoidosa* has the highest survival rate in the environment contaminated with CBM and has a rapid response ability to toxic stimuli [16,25]. The cell viability percentage (Figure S4) is presented in additional material) was above 97.4% for the 20 µg/mL CBM concentration and 74% for the 150 µg/mL CBM concentration.

It is known that the amount of chlorophyll is proportional to the intensity of photosynthesis. In our tests, it was found that the chemical stress generated a gradual fluorescence, inducing in the chlorophyll molecule an inhibition of the photosynthesizing apparatus [57,58].

This was observed by analyzing the fluorescence of chlorophyll. The results indicated that gradual concentrations of CBM gradually inhibit photosynthesis in the chlorophyll molecule [59]. By inhibiting photosynthesis, the mechanism involved in cellular development processes is destroyed. The chemical stress in which the analyzed suspensions were situated generated a gradual fluorescence proportional to the amount of toxin introduced into the bioreactor.

The cyanogenic properties of CBM were investigated, and demonstrated the hydrolysis of CBM to malononitrile, the latter being converted to cyanide. The cyanogenic properties of CBM are due to the fact that the cyanide produced as a metabolite of this substance is extremely toxic [59,60].

We assume that this disturbs the operation of the photosynthesizing apparatus of the *C. pyrenoidosa* microorganism, leading to a reduction in cell growth due to oxidative stress [18]. We assume that for this reason, the amount of chlorophyll in the sample decreases, and the fluorescence emission of chlorophyll changes directly proportional to cell inhibition because chlorophyll “a” is the pigment that participates in the energy transfer to the enzymatic reaction centers in the algal cell [60,61,62,63].

**Figure 9:**
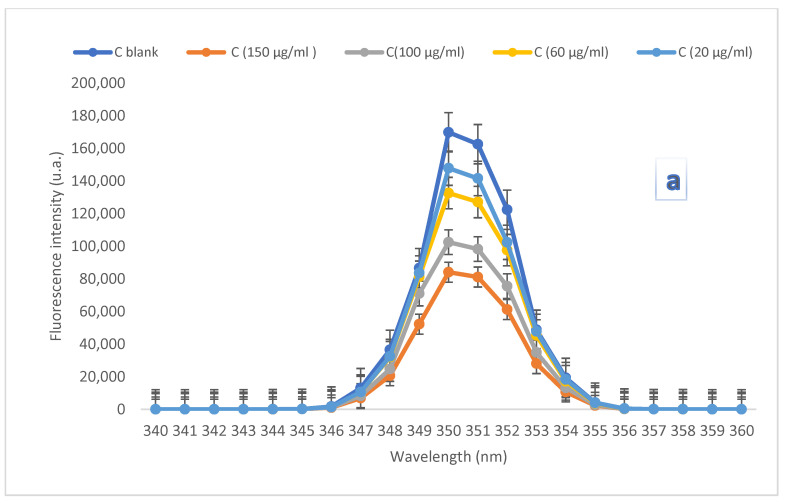

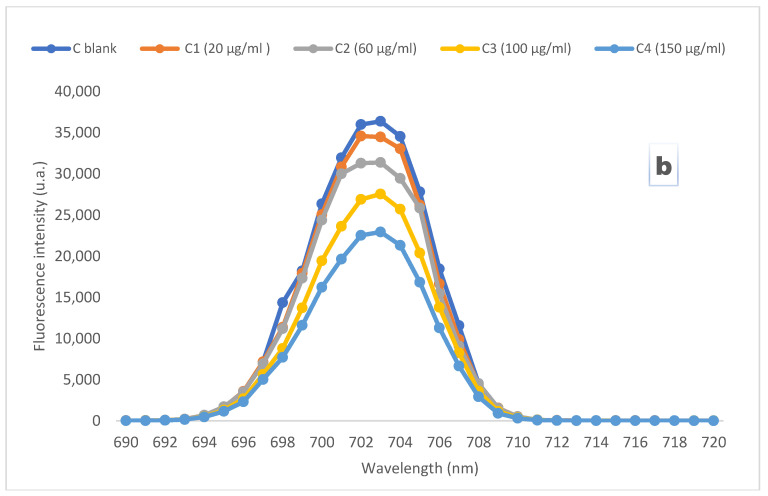
Fluorescence emission in samples incubated for 24 h. Emission (**a**) 350 nm, (**b**) 700 nm. Error bars represent standard deviation (n = 4).

Fluorescence measurements were made after 24 h of sample incubation (Figure 9) [63,64,65]. After analyzing the results obtained regarding the fluorescence emission presented graphically, we obtained values of the gradual fluorescence intensity directly proportional to the concentration of chlorophyll in the samples. The interpretation of the fluorescence spectrum is based on the principle according to which the chlorophyll molecule was hit by electromagnetic radiation and was excited, which generated the jump of an electron from a close orbital to a more distant one. This transition is unstable, and the electron returns to its original position or is given to another molecule. The emission of radiation by the chlorophyll molecule is based on this principle [60,61,62,66,67]. This radiation differs from the radiation that hits the molecule with lower energy and a longer wavelength; this de-excitation process generates the fluorescence of the chlorophyll molecule contained in the algal suspensions.

The fluorescence curves we obtained show that after excitation at a wavelength of 350 nm, we obtained a peak intensity of approximately 175,000 u.a. in the control sample, approximately equal to the intensity obtained after the excitation of the sample containing 20 µg/mL CBM (160,000 u.a.). The sample containing 150 µg/mL CBM had a fluorescence intensity of 80,000 u.a. For the pulse obtained when the sample was excited at 700 nm, we obtained for the sample containing 20 µg/mL CBM a fluorescence intensity of 35,000 u.a. and the sample containing 150 µg/mL CBM had a fluorescence intensity of 22,000 u.a. If we consider the peak fluorescence emission for the control as 100%, we can consider that the fluorescence emission for the sample of 150 µg/mL CBM was 53.5% of the intensity of the control at the wavelength of 350 nm and 55% from the intensity of the control at the wavelength of 700 nm.

## 5. Conclusions

Following the experiments carried out, it was observed that the algae C. pyrenoidosa has a high tolerance to the toxic CBM, the inhibition of cell development generated by different concentrations of CBM measured by oxygen production is small, the curve obtained after measuring the process of algal assimilation and oxygen consumption indicates a moderate toxic action. The values obtained for pH during the adaptation period of the *C. pyrenoidosa* culture were between 6.0 and 6.8, O_2_ had values between 6.5 and 7.0 mg/L, and conductivity of 165–210 µS/cm. The amount of chlorophyll decreases as the CBM concentration in the samples increases. When the chemical stress yield increases, the fluorescence yield decreases. Algae biocoagulation is useful information that could have applicability in the bioremediation of the CBM toxic environment because *C. pyrenoidosa* showed cellular stability in the presence of chemical stress with the formation of conglomerates that protect the cellular structure.

After considering the experimental results, it was concluded that microbiological cultures of *C. pyrenoidosa* are responsive bioindicators of stress conditions and are sensitive to CBM. The sensitivity of algae cells has made them alternative models to microorganisms for biomonitoring studies and assessment of chemical toxicity. When chemicals come into touch with microbes, they use them in their own biochemical processes. In metabolic reactions, organic substances provide carbon and energy for the biochemical processes that provide microorganisms the energy they need to perform their essential functions. Even a concentration of 20 µg/mL CBM produces observable effects on algal biomass, generating toxicity on cells, inhibiting photosynthesis, and, implicitly, cell development. Therefore, ecotoxicity tests could be considered a useful tool not only in laboratory tests but also in hazardous wastewater management. By supplementing the biofauna in the contaminated areas with suspensions of *C. pyrenoidosa* positive results are expected in order to improve the quality of environmental factors affected by the presence of pollutants at concentration levels that represent a significant risk for the environment and human health.

## Data Availability

The data presented in this study is openly available in the public repositories.

## Supplementary Materials

The following supporting information can be downloaded at: https://www.mdpi.com/article/10.3390/bioengineering11060623/s1, Figure S1: FTIR spectra of *Chlorella pyrenoidosa*; Figure S2: FTIR spectra of CBM 100 ppm; Figure S3: FTIR spectra of chlorophyll (Chl); (A)-*Chlorrella* sp. incubated 24 h, before chlorophyll extraction, (B)-*Chlorrella* sp. blank after analysis of Chl, (C) (20 µg/mL ), (D) (60 µg/mL ), (E) (100 µg/mL ), (F) (150 µg/mL; Figure S4: The cell viability percentage after 24 h of incubation. µg/mL.
