# Supplementary material for: Reduction of Oxygen Production by Algal Cells in the Presence of O-Chlorobenzylidene Malononitrile
Source: Bioengineering (Basel). 2024 Jun 18;11(6):623. doi: 10.3390/bioengineering11060623 (PMC11200456; doi:10.3390/bioengineering11060623)
Supplement: Supplementary file 1 [file bioengineering-11-00623-s001.zip › bioengineering-2951456-supplementary.pdf]

bioengineering

MDPI

Article

Reduction of Oxygen Production by Algal Cells in the Presence of O-Chlorobenzylidene Malononitrile

Viorel Gheorghe, Catalina Gabriela Gheorghe *, Daniela Roxana Popovici *, Sonia Mihai, Raluca Elena Dragomir and Raluca Somoghi

Chemistry and Chemical Engineering Department, Petroleum—Gas University of Ploiesti, 39 Bvd. Bucuresti, 100520 Ploiesti, Romania; gheorgheviorel1@gmail.com (V.G.); smihai@upg-ploiesti.ro (S.M.); rdragomir@upg-ploiesti.ro (R.E.D.); r.somoghi@gmail.com (R.S.)
* Correspondence: catalina.gheorghe@upg-ploiesti.ro (C.G.G.); dana_p@upg-ploiesti.ro (D.R.P.)

Supplementary Materials

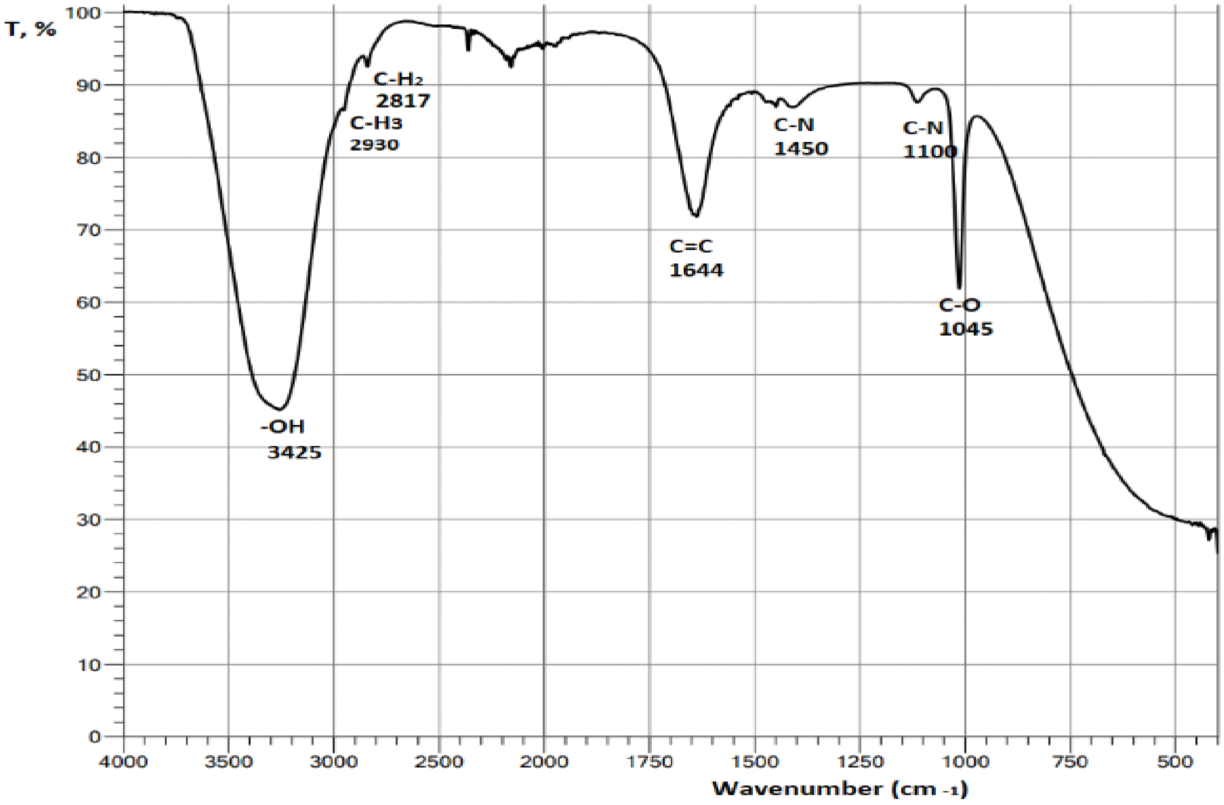

Figure S1. FTIR spectra of *Chlorella pyrenoidosa*.

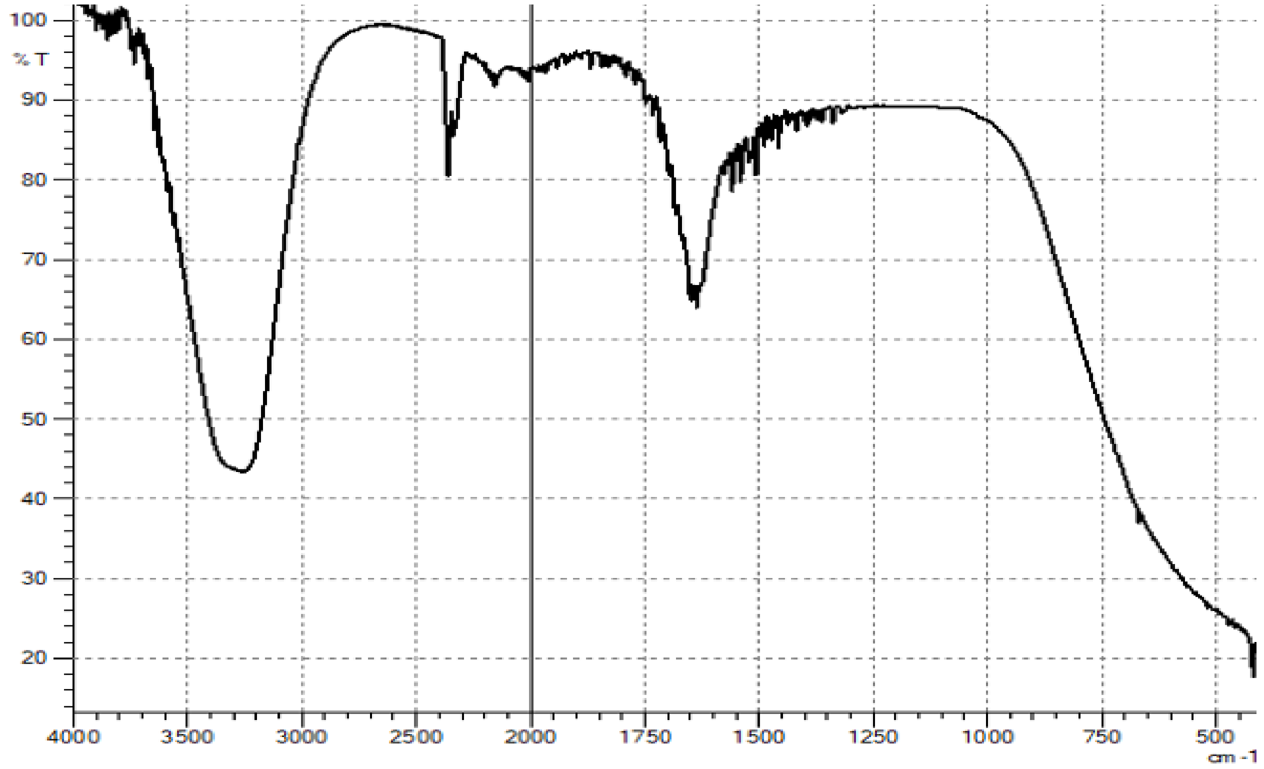

Figure S2. FTIR spectra of CBM 100 ppm.

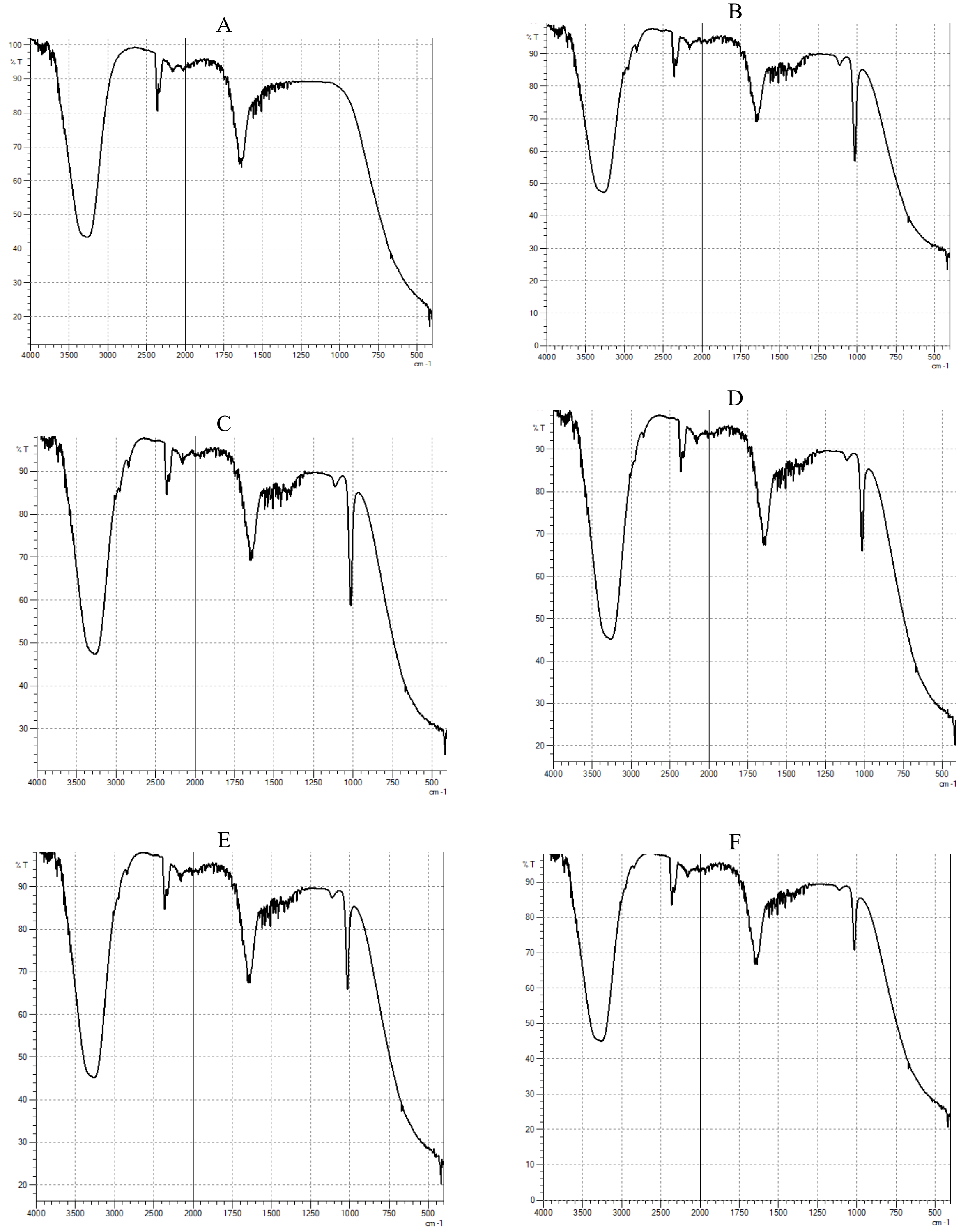

Figure S3. FTIR spectra of chlorophyll (Chl); (**A**) Chlorrella sp. incubated 24 h, before chlorophyll extraction, (**B**) *Chlorrella* sp. blank after analysis of Chl, (**C**) 20 µg/mL, (**D**) 60 µg/mL, (**E**) 100 µg/mL, (**F**) 150 µg/mL.

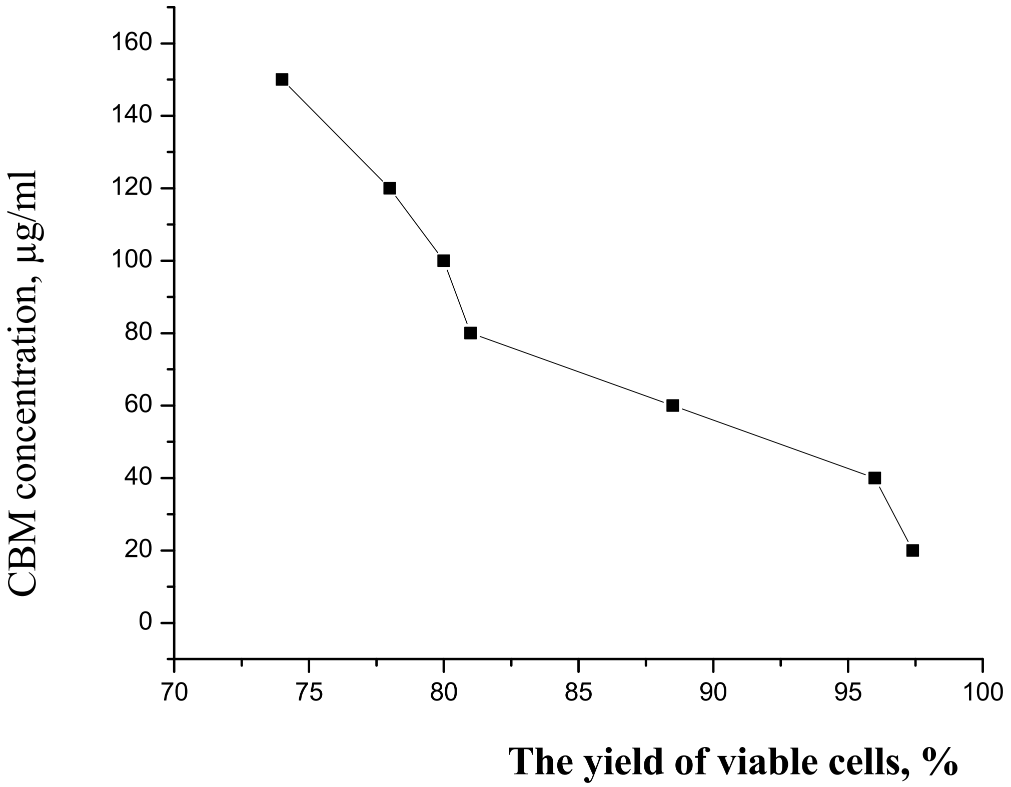

Figure S4. The cell viability percentage after 24 h of incubation. μg/mL.
